# The whole blood phagocytosis assay: a clinically relevant test of neutrophil function and dysfunction in community-acquired pneumonia

**DOI:** 10.1186/s13104-020-05034-0

**Published:** 2020-04-08

**Authors:** J. Reiné, J. Rylance, D. M. Ferreira, S. H. Pennington, I. D. Welters, R. Parker, B. Morton

**Affiliations:** 1 Clinical Sciences Department, Liverpool School of Tropical Medicine (LSTM), Liverpool, UK; 2 Critical Care Department, Royal Liverpool University Hospital, Liverpool, UK; 3 Critical Care Department, Aintree University Hospital NHS Foundation Trust, Liverpool, UK; 4 Lung Health Group, Malawi Liverpool Wellcome Trust Clinical Research Programme, Queen Elizabeth Central Hospital, College of Medicine, P.O. BOX 30096, Chichiri, Blantyre, Malawi

**Keywords:** Neutrophils, Sepsis, Flow cytometry, Phagocytosis, Pneumonia

## Abstract

**Objective:**

To refine and validate a neutrophil function assay with clinical relevance for patients with community-acquired pneumonia (CAP).

**Design:**

Two phase cross-sectional study to standardise and refine the assay in blood from healthy volunteers and test neutrophil phagocytic function in hospital patients with CAP.

**Participants:**

Phase one: Healthy adult volunteers (n = 30). Phase two: Critical care patients with severe CAP (n = 16), ward-level patients with moderate CAP (n = 15) and respiratory outpatients (no acute disease, n = 15).

**Results:**

Our full standard operating procedure for the assay is provided. Patients with severe CAP had significantly decreased neutrophil function compared to moderate severity disease (median phagocytic index 2.8 vs. 18.0, p = 0.014). Moderate severity pneumonia neutrophil function was significantly higher than control samples (median 18.0 vs. 1.6, p = 0.015). There was no significant difference between critical care and control neutrophil function (median 2.8 vs. 1.6, p = 0.752).

**Conclusions:**

Our whole blood neutrophil assay is simple, reproducible and clinically relevant. Changes in neutrophil function measured in this pneumonia cohort is in agreement with previous studies. The assay has potential to be used to identify individuals for clinical trials of immunomodulatory therapies, to risk-stratify patients with pneumonia, and to refine our understanding of ‘normal’ neutrophil function in infection.

## Introduction

Community-acquired pneumonia (CAP) is a major cause of morbidity and mortality. Disease results from both the bacterial infection, and the ensuing inflammatory host response, which, during early infection, is dominated by neutrophils. Neutrophil dysfunction is well recognised in patients with CAP and sepsis [1, 2]. Currently, there are no clinically validated laboratory tests of neutrophil function; this limits the development and testing of potential immunomodulatory therapies [3]. Personalised approaches to immunomodulatory drug administration could enhance positive outcomes and reduce the risk of potentially harmful adverse-effects [2]. We aimed to refine and validate a flow-cytometric whole blood assay of neutrophil phagocytic function using intraphagosomal reporter beads [4, 5]. Potential advantages of this assay are direct measurement of relevant biological activity, minimal sample processing and the potential to become a ‘near-patient’ test with sample to result time of less than 4 h.

## Main text

### Methods

We recruited healthy volunteers and hospital patients in a two-phase study. The North West-Haydock Research Ethics Service (UK) approved this study (15/NW/0869). In phase one, we refined our standard operating procedure (SOP) using blood from healthy adult (> 18 years old) volunteers. In phase two, we recruited adult patients within 48 h of diagnosis of severe and moderate CAP (n = 16; defined by NICE guidelines, CG191: 2014 [6] who were admitted to critical and ward level care respectively (n = 15). A control group of outpatients with chronic stable respiratory disease (no acute inflammatory disease, n = 15) were recruited for comparative purposes (July 2016–April 2017). Hospital patients were recruited from Aintree University Hospital and Royal Liverpool University Hospital, Liverpool, United Kingdom. Participants were excluded if diagnosed with an immunocompromising condition or therapy (including HIV infection, malignancy and long term (> 2 weeks) corticosteroid treatment), pregnant, requiring renal replacement therapy, unable to provide informed consent.

Citrated blood samples were transported to the laboratory and processed within 2 h of venepuncture. Finalised methods for the whole blood phagocytosis assay were refined based on previously published work [4, 5]. Samples were acquired using a BD LSR II flow cytometer equipped with three lasers (405, 488, and 633 nm; Becton–Dickinson, USA), using compensation matrices derived from commercial beads (Becton–Dickinson, USA). Data were acquired using FACS Diva software (version 6.1, BD Biosciences, USA) and analysed using FlowJo software (version 10, Tree Star, USA). A sequential gating strategy was used to identify the neutrophil according to light scatter and the expression of CD16 (Additional file 1: Figure S1). Neutrophil oxidation ratio (OR) was calculated as the mean fluorescence intensity (MFI) of the reporter fluorophore (FITC) divided by the MFI of the calibrator fluorophore (Pacific Blue). To confirm the interaction of blood neutrophils and the intraphagosomal reporter beads during the assay, illustrative confocal images and time-lapse video were also provided in Additional file 1: Figure S2, Video S1; Additional file 2.

Neutrophil phagocytic index (PI) was calculated as the number of neutrophils associated with reporter beads divided the total neutrophil number multiplied by the OR (Additional file 1: Figure S1). Statistical analysis was performed using Stata 13.1 for Mac (StataCorp LP, 2015); data were tested for normality using the Shapiro–Wilk test and analysed using parametric and non-parametric tests, as appropriate.

### Results

We analysed whole blood samples from 16 patients with severe CAP admitted to critical care, 15 patients with moderate severity CAP admitted to hospital wards and 15 patients with chronic, non-inflammatory and stable respiratory disease. Patient demographics, illness severity, microbiological status and outcome data were also recorded (Table 1). Median venepuncture to result (completed sample acquisition) time was 305 (IQR 261–328) min, including median 91 (IQR 49–118) min transport time between sampling and arrival in the laboratory. There were 45 min hands on time (i.e. pre-analytical interaction, reagent and sample preparation and loading, in-process interaction, post-analytical interaction and maintenance), per patient (sample batching promoted reductions).

**Table 1:** Clinical findings of ITU and ward patients in comparison to healthy control

	ITU (n = 16)	Ward (n = 15)	Outpatient (n = 15)
Female	9	9	9
Age^a^	57 (38–68)	64 (38–80)	49 (43–63)
SOFA^b^	9 (4–12)	1 (0–3)	N/A
CURB-65^b^	3 (3–4)	3 (1–4)	N/A
Mortality (28 day)	2	0	0
Hospital LOS	18 (9–26)	6 (2–11)	N/A
ICU LOS	10 (4-17)	N/A	N/A
Respiratory support	Invasive ventilation = 11	Room air = 11	Room air = 15
	Face mask CPAP = 3	Nasal specs = 4	
	Face mask high flow = 2		
Microbiology	No growth = 9	No growth = 15	N/A
	*S. pneumoniae *= 3		
	*E. coli *= 1		
	Influenza = 1		
	Metapneumovirus = 2		
Beta-lactam antibiotic (glycopeptide synthase inhibitor if penicillin allergic)	Benzylpenicillin = 4	Benzylpenicillin = 9	N/A
	Amoxicillin = 2	Amoxicillin = 1	
	Tazocin = 9	Tazocin = 4	
	Teicoplanin = 1		
Adjunct antibiotic	Clarithromycin = 13	Clarithromycin = 11	
	Clindamycin = 1	Ciprofloxacin = 1	
	Gentamicin = 1		
	Metronidazole = 1		
Antiviral	Oseltamavir = 3	Oseltamivir = 1	

ITU, severe sepsis patients; ward, sepsis patients; outpatient indicates healthy control; SOFA, sequential organ failure assessment score; CURB-65, pneumonia severity score based on confusion, urea nitrogen > 20 mg/dL, respiratory rate > 30 breaths/min, heart beat: Systolic BP < 90 mmHg or diastolic BP < or = 60 mmHg) and Age ≥ 65; ICU, intensive care unit; LOS; CPAP, continuous positive airway pressure; N/A, not applied

^a^Median (range)

^b^Median (25th–75th percentile)

**Fig. 1:**
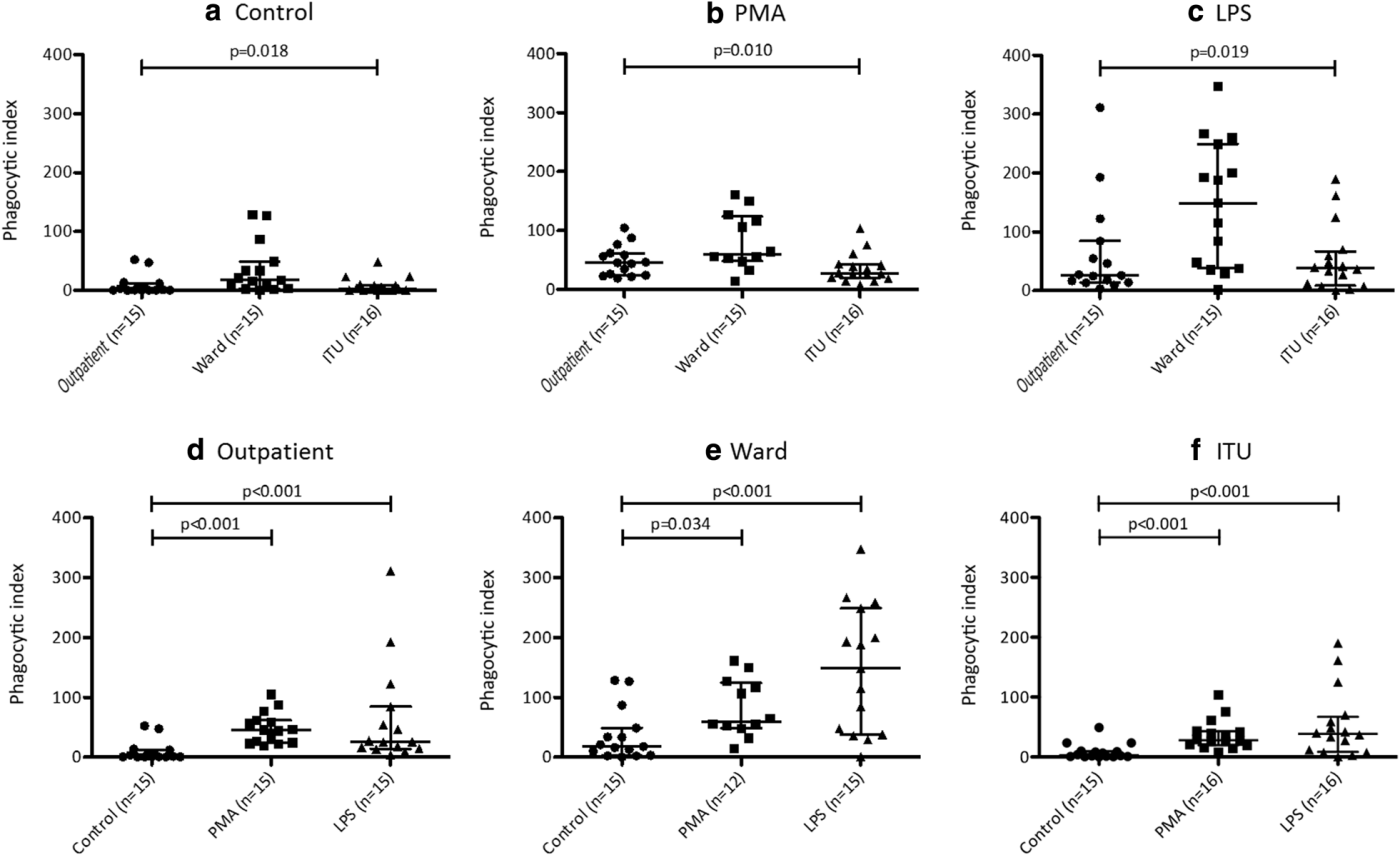
Neutrophil phagocytic index in whole blood samples taken from patients with severe and mild/moderate severity community-acquired pneumonia (“ITU” and “ward” respectively), and respiratory outpatients with no acute inflammatory disease. Phagocytic index was calculated after 45 min incubation. Neutrophils were identified according to the expression of CD16. Neutrophil oxidation ratio (OR) was calculated as the mean fluorescence intensity (MFI) of the reporter fluorophore (FITC) divided by the MFI of the calibrator fluorophore (Pacific Blue). Neutrophil phagocytic index (PI) was calculated as the number of neutrophils associated with reporter beads divided the total neutrophil number multiplied by the OR. Each dot represents data collected from a single volunteer. Samples were incubated at 37 °C, with shaking, in the presence of either **a** a vehicle control, **b** PMA or **c** LPS. Comparisons were made using Kruskall–Wallis (3 groups) and Mann–Whitney U (2 groups) tests, as appropriate

Neutrophil phagocytic index (PI) was significantly higher in unstimulated blood samples taken from ward patients (moderate pneumonia) compared to critical care (severe pneumonia) patients [median 18.0 (IQR = 3.0–48.7) vs. 2.8 (IQR = 2.8–9.3) p = 0.014] and outpatients [median 18.0 vs. 1.6 (IQR = 0.7–12.0), p = 0.015]. However, there was no significant difference between critical care and outpatients (Fig. 1a; median 2.8 vs. 1.6, p = 0.752). For each group, PI value was significantly increased after neutrophil stimulation with phorbol myristate acetate (PMA) and lipopolysaccharide (LPS) (Fig. 1d–f). However, response to these positive controls was attenuated in critical care compared to ward patients. (Figure 1b, c) suggesting reduced maximal responses in the former group.

### Discussion

We present a refined flow cytometry assay [5], clinically relevant method for measuring neutrophil function in blood taken from patients with CAP ex vivo. Our technique avoids pre-processing of blood, is simple to perform and reproducible. It directly measures phagocytic association with, and oxidation of neutrophils (representative of host/pathogen interaction) and can deliver results within 4 h of venepuncture. This assay can detect attenuated phagocytic function in patients with severe compared to mild-moderate community acquired pneumonia.

Pneumonia is the most frequent source of sepsis requiring critical care admission and induces disproportionate rates of mortality and morbidity compared to other severe infections [7]. In light of increasing antimicrobial resistance, there is increased pressure to investigate potential immunomodulatory agents, such as GM-CSF and interferon gamma [3], using a personalised approach to therapy [2]. To date, only indirect cell surface markers of phagocyte function have been employed in clinical trials [8]. Previously described zymosan neutrophil assays require cell purification (removing the inflammatory milieu) and are time consuming [9]. Other, recently described, whole blood functional assays exist [10]; however, these have not been studied in the context of sepsis. Direct comparison of these functional assays would be useful. Our approach promotes direct measurement of phagocytic function and will be used as the primary pharmacodynamic outcome measure for a planned first-in-human clinical trial with P4 peptide [5].

In summary, we present a simple, clinically relevant assay that can be used to measure neutrophil phagocytic function in patients who present clinically with community-acquired pneumonia. This approach has the potential to be applied in a wider clinical context to measure neutrophil function in inflammatory disease and potentially direct immunomodulatory therapies.

## Limitations

Due to the relatively small cohort size, we were unable to study factors predictive of poor neutrophil function (e.g. microbiological status), or relationship to patient outcomes. Future large-scale studies could assess these in cohorts suffering from pneumonia and undifferentiated sepsis disease states. Results could potentially be used to inform ‘normal-range’ neutrophil function and triage patient admission decisions (e.g. promote direct critical care admission) and/or direct targeted immunomodulatory therapies. Future investigations should seek to age-match outpatient controls to mitigate for this potential confounding factor in our study.

## Supplementary information

**Additional file 1.** Figures S1-S2, Video S1 caption, and standard operation procedure.

**Additional file 2.** Video S1.

## Data Availability

The datasets generated during and/or analysed during the current study are available from the corresponding author on reasonable request.

## Abbreviations

CAP: Community-acquired pneumonia
MFI: Mean fluorescence intensity
OR: Oxidation ratio
PI: Phagocytic index
PMA: Phorbol-myristate-acetate
LPS: Lipopolysaccharide
PMT: Photomultiplier tubes
