# Supplementary material for: The whole blood phagocytosis assay: a clinically relevant test of neutrophil function and dysfunction in community-acquired pneumonia
Source: BMC Res Notes. 2020 Apr 8;13:203. doi: 10.1186/s13104-020-05034-0 (PMC7140487; doi:10.1186/s13104-020-05034-0)
Supplement: Supplementary file 1 — Additional file 1. Figures S1-S2, Video S1 caption, and standard operation procedure. [file 13104_2020_5034_MOESM1_ESM.pdf]

Additional DATA

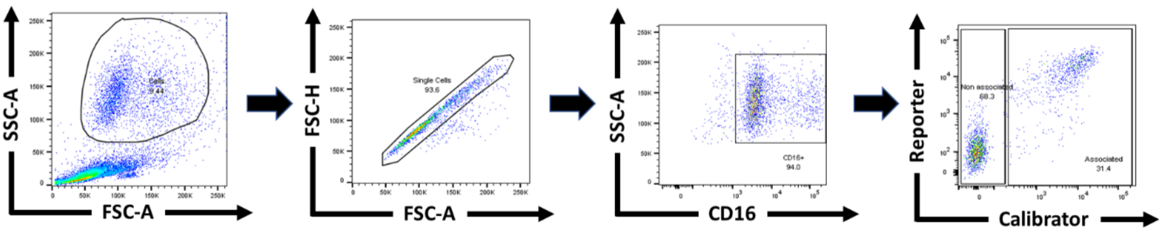

Figure S1 Neutrophils were identified according to surface expression of CD16 and light scatter characteristics. Neutrophil oxidation ratio (OR) was calculated as the mean fluorescence intensity (MFI) of the reporter fluorophore (FITC) divided by the MFI of the calibrator fluorophore (Pacific Blue). Neutrophil phagocytic index (PI) was calculated as the number of neutrophils associated with reporter beads divided the total neutrophil number multiplied by the OR.

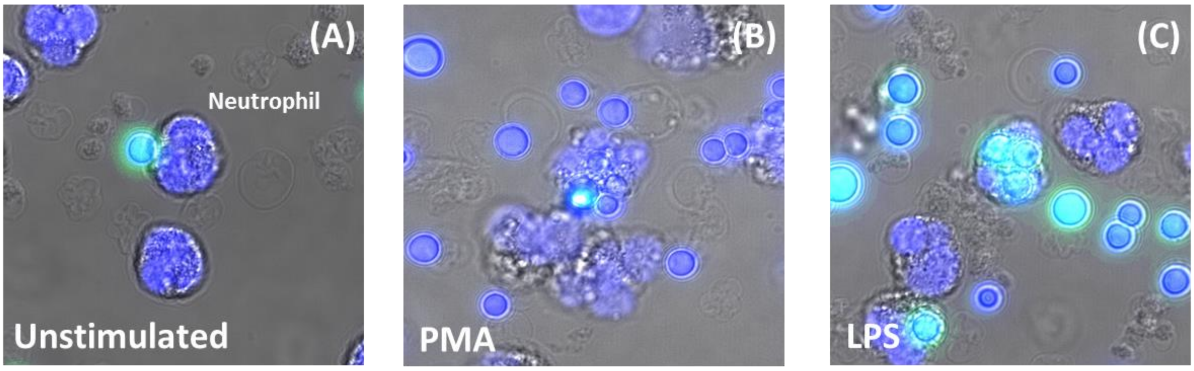

Figure S2 Representative confocal imaging of neutrophil and intraphagosomal reporter bead interaction. Neutrophils were isolated by differential centrifugation, stained with Hoechst, and placed in culture at 37°C, with intraphagosomal reporter beads in the presence of either (A) Unstimulated, (B) PMA or (C) LPS.

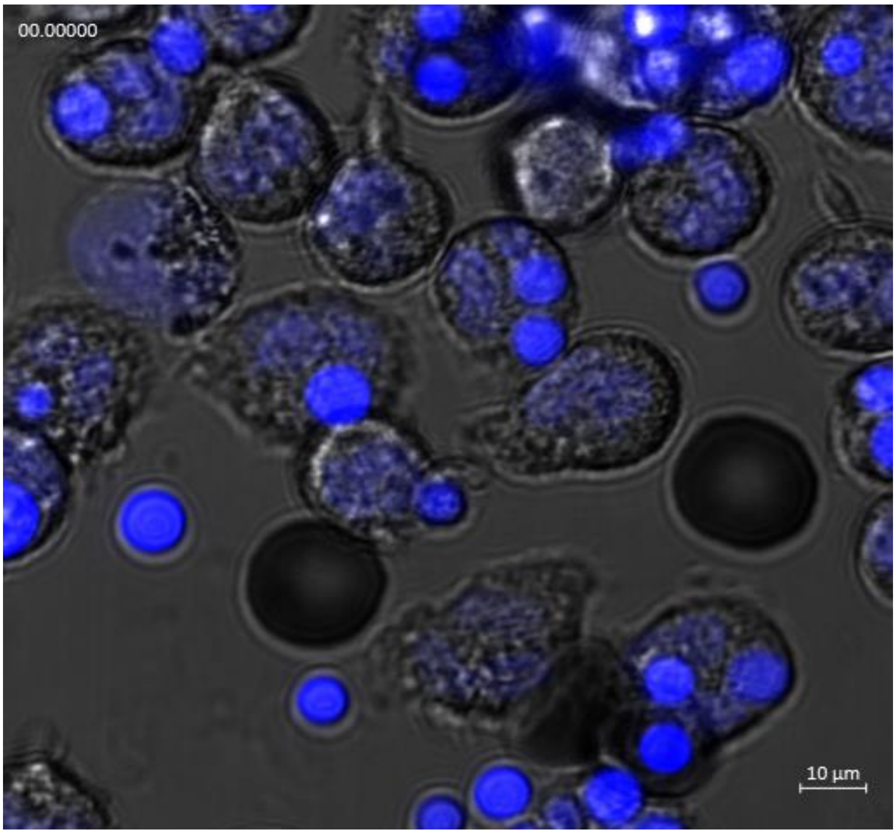

Video S1 Time-lapse video analysis of the whole blood phagocytosis assay by confocal microscopy. The phagocytosis of the beads occurs within a small number of steps; (1) attachment of the bead to the surface of the neutrophil presumably by IgG receptors, (2) Uptake of the bead, (3) internalization of the bead into a phagolysosome (4) oxidation of the bead.

Additional data: Whole Blood Phagocytosis Assay Standard Operation Protocol (SOP)

This document contains the methodological principles of the standardization and refinement of the phagocytosis and oxidative burst in whole blood samples by flow cytometry. The accurate analysis of the phagocytosis requires knowledge about the type of target populations, the fluorochromes/dyes involved and the optimization of technical challenges during the assay.

Here, we describe a simple, fast, accurate and highly reproducible assay to measure the neutrophil function by flow cytometry to be used as a biomarker in sepsis patients. One of the additional goals in our study is to define a preliminary SOPs to establish a standardized instrument setting that would allow reproducible (identical or highly reproducible) measurements of the whole blood phagocytosis assay using the same cytometer or in different instruments.

Instrument setup:

Instrument performance (BD LSR II Cytometer):

1. Start up the instrument. Prime twice.
2. Allow 30 minutes for the lasers to stabilize.
3. Create a new experiment.
4. Create a worksheet with all the necessary dot plots, histograms and statistics (please see proposed gating strategy section)
5. Acquire Cell Setup Tracking beads (CST) or equivalent beads to monitor the instrument parameters and the laser status before to start any acquisition.
6. Label your samples tubes. Acquire and record the samples

Settlement of the instrument settings for light scatter measurements (FSC and SSC-A):

- PMT voltages will be adjusted to forward scatter (FSC-A) and sideward scatter (SSC-A) gated on bead target population (neutrophils). Acquire cells, gate lymphocytes and adjust fine-tune FSC and SSC voltage to reach the following mean target values:
    - FSC-A values around 45,000-70,000
    - SSC-A values around 11,000-15,000
    - Threshold in FSC around 5,000
- Acquire and record 50,000 events to verify settings, readjust if necessary. The discrimination of all the leukocyte populations must be clear and resolute. The populations cannot be hidden in the axis.
- Inclusion of FSC-H parameter is mandatory to allow the discrimination of doublets in an FSC-Area (FSC-A) versus FSC-Height (FSC-H) bivariate plot, contributing to the precision of the results.

Fluorescence compensation standards:

- The emission wavelength of the reporter (OxyBurst) and the calibrator (Alexa 405 SE) attached to the beads are in different lasers with different emission wavelengths, so there is not spectral overlap between them. For the beads no compensation is required.
- The spectral properties of the Oxyburst are the following: Ex/Em wavelength: 492-495/517-527 nm. Excitation laser (488nm – Blue). Similar spectral dye: FITC. The spectral properties of the Alexa 405 SE are the following: Ex/Em: 400/424nm. Excitation laser (405 nm – Violet). Similar spectral dye: Pacific Blue.
- The CD16-APC antibody doesn’t require compensation with the beads because the emission wavelength of APC *per se* is in another spectral emission (Ex/Em wavelength 633/650-670nm).

Validation controls of the assay:

- Unstained whole blood sample to define the background, the auto-fluorescence and the establishment of the light scatter parameters previously described.
- Positive control: Cells stimulated with lipopolysaccharide (LPS) and Phorbol-myristate-acetate (PMA) independently during 45 minutes at 37ºC protected from light (see experimental procedure).
- Control of the beads. Acquisition of beads not mixed with the whole blood sample (see experimental procedure) as a negative control.

Proposed gating strategy: See Figure S1

Experimental procedure:

Phagocytosis assay:

1. Add 100µL of human peripheral blood from a NC3 blood tube (sodium citrate anticoagulant) into the Facstubes that already contains the 120µL solution of the beads.
2. Mix gently. Use a vortex.
3. Incubate with shaking (300 rpm) at 37ºC protected from light during the different time kinetics. The mixing speed is important in the phagocytosis assay. Continuous mixing of the samples increases the contact of the target population (neutrophils) with the beads allowing an increase of the phagocytosis.
4. Once the incubation time kinetics is finished, to stop the reaction of phagocytosis, put all the samples on ice immediately (at 4ºC). The phagocytosis reaction will be completely stopped. Keep the samples on ice for 5 minutes protected from light.
5. Add 5µL of the labelled antibody per test (CD16 APC) to the samples on ice. Incubate the antibodies for 15 minutes on ice protected from light.

6. Once the incubation is finished for all the samples. Add directly 2mL of FACS Lysing solution 1X [FACS Lysing solution 10x is diluted 1/10 -vol/vol- in distilled water (H_20d)]
7. Mix gently. Use the vortex.
8. Incubate 20 minutes on ice (at 4°C) protected from light.
9. Centrifuge for 5 minutes at 1500 rpm.
10. Discard the supernatant.
11. Add 2 mL of washing buffer PBS 1X to the cell pellet.
12. Mix gently. Use the vortex. Be aware the sample is completely homogeneous.
13. Centrifuge for 5 minutes at 1500 rpm.
14. Discard the supernatant.
15. Add 250µL of PBS 1X per tube.
16. Mix gently. Acquire the samples immediately or within 2 hours after the staining/assay procedure. Note: If the samples are not acquired immediately in the flow cytometer, store at 4°C until measurement is performed.

Limitations:

- Blood samples should be stored at 18-22°C and tested immediately when they are received. Note: If the samples are received immediately after the venepuncture please wait 30 minutes before to start and leave the samples in a roller sampler to keep it homogenous during these 30 minutes.
- Results obtained by flow cytometry may be erroneous if the cytometer laser is misaligned or the gates are improperly set. Please be aware about the instrument performance of the instrument before to start the acquisition.
- It is advisable to acquire the samples to the cytometer as soon as possible to optimize the results. Non-viable cells may stain non-specifically.

Considerations and future actions for this protocol:

- The inclusion of novel extracellular markers combined to the beads could be easily adapted and focused for a deeper understanding and higher detailed identification of the major myeloid lineages for a better clinical input. The inclusion of CD66b (degranulation marker) combined with the CD16 will be useful for a better discrimination of mature and immature neutrophils. The inclusion of CD14 and HLADR combined with CD16 will allow to discriminate the phagocytic capabilities of the monocytes at different stages of monocytic maturation. The addition of new markers will be always limited by the optical configuration itself of the instrument where the assay is going to be acquired.
- One of the greatest advantages and benefits of this assay is its flexibility and quick assessment, not only in adults. This assay can be extrapolated to paediatric cohorts easily because the volume of whole blood required is 100µL (2-3 drops) per test providing very useful information about the myeloid function capabilities in different clinical conditions.
- The second advantage is its relatively low cost of the assay that would facilitate its introduction in under develop countries providing an additional clinical tool based on the assessment of the phagocytic capacity of the myeloid populations as biomarker for sepsis or any other clinical condition.
